# Charge-Ordering and Structural Transition in the New Organic Conductor δ′-(BEDT-TTF)_2_CF_3_CF_2_SO_3_

**DOI:** 10.1021/acs.jpcc.1c09458

**Published:** 2022-01-25

**Authors:** Iwona Olejniczak, Bolesław Barszcz, Pascale Auban-Senzier, Harald O. Jeschke, Roman Wojciechowski, John A. Schlueter

**Affiliations:** † Institute of Molecular Physics, Polish Academy of Sciences, Smoluchowskiego 17, 60-179 Poznań, Poland; ‡ Laboratoire de Physique des Solides, Université Paris-Saclay, UMR 8502 CNRS, Université Paris-Sud, Orsay 91405, France; ¶ Research Institute for Interdisciplinary Science, Okayama University, Okayama 700-8530, Japan; § Department of Molecular Physics, Faculty of Chemistry, Technical University of Łódź, Żeromskiego 116, 90-924 Łódź, Poland; ∥ Materials Science Division, Argonne National Laboratory, Argonne, Illinois 60439, United States; ⊥ Division of Materials Research, National Science Foundation, 2415 Eisenhower Avenue, Alexandria, Virginia 22314, United States

## Abstract

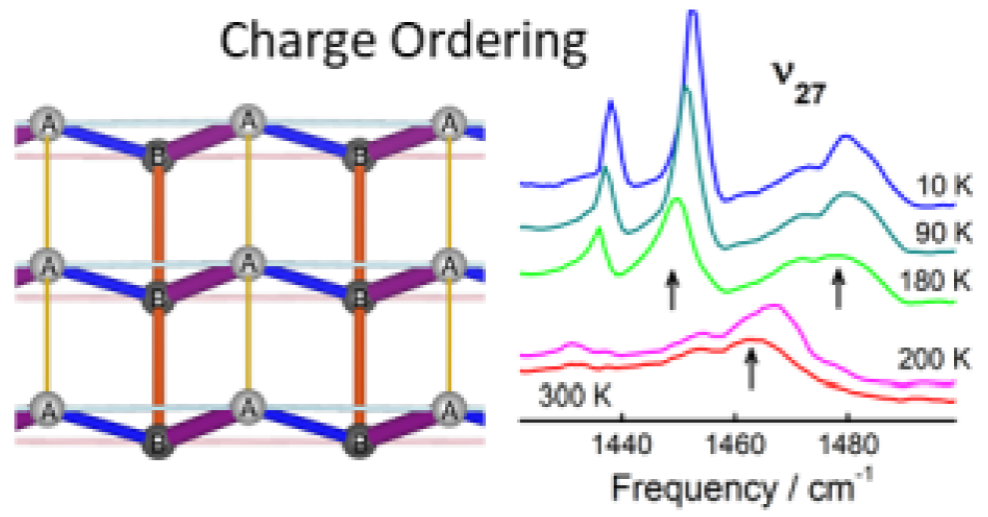

We report structural, transport, and optical properties and electronic structure calculations of the δ′-(BEDT-TTF)_2_CF_3_CF_2_SO_3_ (BEDT-TTF = bis(ethylenedithio)tetrathiafulvalene) organic conductor that has been synthesized by electrocrystallization. Electronic structure calculations demonstrate the quasi-one-dimensional Fermi surfaces of the compound, while the optical spectra are characteristic for a dimer–Mott insulator. The single-crystal X-ray diffraction measurements reveal the structural phase transition at 200 K from the ambient-temperature monoclinic *P*2_1_/*m* phase to the low-temperature orthorhombic *Pca*2_1_ phase, while the resistivity measurements clearly show the first order semiconductor-semiconductor transition at the same temperature. This transition is accompanied by charge-ordering as it is confirmed by splitting of charge-sensitive vibrational modes observed in the Raman and infrared spectra. The horizontal stripe charge-order pattern is suggested based on the crystal structure, band structure calculations, and optical spectra.

## Introduction

Layered organic charge transfer salts based on the BEDT-TTF donor molecule are known to display competing ground states depending on the details of molecular structure and strength of the onsite and intersite electronic Coulomb correlations.^1−4^ The (BEDT-TTF)_2_*X* salts, where *X* is a monovalent anion, are characterized by a variety of dimerized stacking arrangements of the conducting BEDT-TTF layer traditionally labeled using Greek letters, such as α, β, δ, κ, and θ.^3,5−7^ Strongly dimerized compounds described with half-filled conduction bands usually display magnetically ordered Mott insulating states or superconductivity at low temperature.^8^ In contrast, less dimerized quarter-filled materials characterized with significant intersite Coulomb repulsion exhibit charge-ordered (CO) localization,^9,10^ recently suggested to take part in the appearance of electronic ferroelectricity in the κ-(BEDT-TTF)_2_*X* family of materials.^11^

Among weakly dimerized charge transfer salts there are those characterized by an unique δ-type arrangement of the donor molecules that are twisted with respect to the stacking axis.^7^ β-(BEDT-TTF)_2_PF_6_,^12,13^ classified as the β-phase for historical reasons,^7^ is an early discovered member of this group. The BEDT-TTF salts that belong to the relatively small δ structural family are confirmed to have charge order states at low temperature.^14−17^ Correspondingly, δ-phase polymorphs based upon BPDT-TTF [bis(propylenedithio)tetrathiafulvalene] are characterized by CO insulating ground states.^18^ In the present paper we explore the appearance of the CO phase in δ′-(BEDT-TTF)_2_CF_3_CF_2_SO_3_, a new δ-type material based on BEDT-TTF and the pentafluoroethylsulfonate CF_3_CF_2_SO_3_^–^ anion.^19^

δ′-(BEDT-TTF)_2_CF_3_CF_2_SO_3_ belongs to the family (BEDT-TTF)_2_RR′ SO_3_, where R = SF_5_ and CF_3_ and *R*′ = CH_2_, CF_2_, CHF, CHFCF_2_, and CH_2_CF_2_, of entirely organic conductors bringing attention to a highly tunable anion that can be easily modified prior to the material synthesis.^20,21^ In this group of materials, β″-(BEDT-TTF)_2_SF_5_CH_2_CF_2_SO_3_ has been investigated as an example of superconductivity mediated by CO fluctuations.^22−25^

Recently, it has been reported that a β′-(BEDT-TTF)_2_CF_3_CF_2_SO_3_ dimer Mott insulator that is characterized by quasi-one-dimensional electronic structure undergoes a transition to an unusual interlayer charge-ordered phase accompanied by significant lattice distortion.^26^ In the present study, we use the X-ray diffraction, transport, infrared, and Raman measurements combined with *ab initio* based electronic structure calculations in order to examine the physical properties of δ′-(BEDT-TTF)_2_CF_3_CF_2_SO_3_ that features the same chemical composition but different molecular structure of the conducting layer compared to β′-(BEDT-TTF)_2_CF_3_CF_2_SO_3_. In particular, our results indicate similar dimer–Mott insulating properties at room temperature but demonstrate different low temperature behavior, with the structural phase transition at about 200 K to a charge-ordered phase stabilized within the conducting layer.

## Synthesis, Theoretical Methods, and Experimental Section

BEDT-TTF was prepared as previously described^27,28^ and was recrystallized from chloroform (Aldrich). Prior to use, 1,1,2-trichloroethane (TCE, Fluka) was distilled from phosphorus pentoxide (Aldrich) and filtered through a column containing neutral alumina. Tetrahydrofuran (THF, Aldrich) was distilled from sodium/benzophenone. 12-Crown-4 (Acros) was used without further purification. Lithium pentafluoroethanesulfonate, Li(CF_3_CF_2_SO_3_), was prepared as previously described.^29^ PPN(CF_3_CF_2_SO_3_) [PPN^+^ = bis(triphenylphosphoranylidene)ammonium] was prepared through a metathesis reaction of (PPN)Cl with Li(CF_3_CF_2_SO_3_). (PPN)Cl (Aldrich, 5.57 g, 9.71 mmol) was dissolved in 950 mL of water. Separately, Li(CF_3_CF_2_SO_3_) (2.0 g, 9.71 mmol) was dissolved in 50 mL of water. The two solutions were combined, with the precipitation of PPN(CF_3_CF_2_SO_3_) as a white powder taking place. This solid was recrystallized from acetone/diethyl ether, resulting in a white crystalline solid. Anal. Calcd for C_38_H_30_P_2_N_1_S_1_O_3_F_5_: C, 61.87; H, 4.10; N, 1.90. Found: C, 61.59; H, 3.99; N, 1.86. Mp: 176–178 °C. Black rod-like crystals of δ′-(BEDT-TTF)_2_CF_3_CF_2_SO_3_ were grown by using the previously described electrocrystallization techniques.^21,30,31^ Li(CF_3_CF_2_SO_3_) (60 mg) and 12-crown-4 (10 mg) were added to 15 mL of TCE and stirred for several minutes. This solution was divided between the two chambers of an H-cell. BEDT-TTF (10 mg) was loaded into the anode chamber. A current density of 0.10 μA/cm^2^ was initially applied and gradually increased over a period of 1 week to 0.25 μA/cm^2^, at which time crystallization of black crystals commenced on the electrode surface. Crystals were grown at 25 °C on platinum wire electrodes for a period of 27 days.

High quality crystals were glued to the tip of glass fiber and mounted on a Bruker APEX II 3-circle diffractometer equipped with an APEX II detector. Data were collected at 300(2) K and 100(2) K, with temperature control achieved through use of an Oxford Cryostream 700 Plus LT Device. The data collection was carried out using Mo Kα radiation (λ = 0.71073 Å) with a frame exposure time of 30 s. The raw intensity data were corrected for absorption (SADABS^32^). The structure was solved and refined using SHELXTL.^33^ A direct-method solution was calculated, which provided most of atomic positions from the electron density map. Full-matrix least-squares/difference Fourier cycles were performed, which located the remaining atoms. All non-hydrogen atoms were refined with anisotropic displacement parameters. Hydrogen atoms were placed in ideal positions and refined as riding atoms with relative isotropic displacement parameters. Structural and refinement parameters are provided in Table 1. Five hemispheres of data were collected with 0.30° ω scans and a detector distance of 60 mm. Data to a resolution of 0.77 Å were considered in the reduction using a rod-like crystal of dimensions 0.50 × 0.10 × 0.10 mm^3^. Unit cells were determined as a function of temperature by collecting 50 frames of 0.30° ω scans at three different settings of ϕ for each temperature set point. Temperature was first cooled from 300 to 100 K in 10° steps with a cooling rate of 1 K/min. Equivalent data were then collected by warming the crystal over the same temperature range.

**Table 1:** Crystal data and Structure Refinement of δ′-(BEDT-TTF)_2_CF_3_CF_2_SO_3_

formula	C_22_H_16_F_5_O_3_S_17_	
*M*_W_	968.37	
phase	δ′	
morphology	rod	
cryst syst	monoclinic	orthorhombic
space group	*P*2_1_/m	*Pca*2_1_
*a*/Å	6.6604(2)	14.4203(4)
*b*/Å	35.4445(9)	6.59590(10)
*c*/Å	14.8807(4)	35.1567(9)
α/deg	90	90
β/deg	92.541(1)	90
γ/deg	90	90
*V*/Å^3^	3509.50(17)	3343.92(14)
*Z*	4	
*D*_*c*_/g cm^3^	1.833	1.924
μ/mm^–1^	1.101	1.156
*F*(000)	1956	
*R*(int)	0.0303	0.0224
total reflns	48759	44231
unique reflns	11852	11363
*I* > 2σ(*I*)	8210	11072
*R*(*F*_0_), *R*_*W*_(*F*_0_^2^)^a^	0.1239, 0.4250	0.0210, 0.0542
refined param.	358	425
*T*/K	300(2)	100(2)

a *R*(*F*_0_) = ∑∥*F*_0_|−|*F*_*c*_∥/∑|*F*_0_|, *R*_*W*_(*F*_0_^2^) = [∑*w*(|*F*_0_^2^| – |*F*_*c*_^2^|)^2^/∑*wF*_0_^2^]^1/2^.

Crystallographic data for the δ′-(BEDT-TTF)_2_CF_3_CF_2_SO_3_ structure at 100 and 300 K has been deposited in CIF format with the Cambridge Crystallographic Data Centre with CCDC numbers 2117993 and 2117992, respectively. Copies of this data can be obtained free of charge.^34^

Calculations were performed on the 100 K crystal structure of δ′-(BEDT-TTF)_2_CF_3_CF_2_SO_3_ reported in this paper. The room temperature structure has not been used due to disorder in both anions and the BEDT-TTF molecules. We perform electronic structure calculations using the full potential local orbital (FPLO) code^35^ with the generalized gradient approximation functional in its Perdew–Burke–Ernzerhof (PBE) form.^36^ We employ projective Wannier functions within FPLO^37^ to determine the tight binding Hamiltonian for the band arising from the HOMO level of BEDT-TTF.

In order to measure the electrical resistivity, annular gold pads were evaporated onto the surface of the single crystals in order to improve the quality of the contacts, and gold wires were glued with silver paste on those pads. The temperature dependence of the resistivity was measured in a homemade cryostat equipped with a 4 K pulse-tube at a cooling or warming rate of 0.5–0.8 K/min. The resistance was measured at four points with an applied current *I*_*dc*_ = 0.5 μA using a Keithley 2400 sourcemeter.

Infrared measurements were performed using two rod-like samples of δ′-(BEDT-TTF)_2_CF_3_CF_2_SO_3_, a 1.7 × 0.6 × 0.5 mm^3^ sample A, and a 1.5 × 1.5 × 1.0 mm^3^ sample B. The optical axes were resolved based on the anisotropy at 300 K. For the sample A we probed two mutually perpendicular directions within the conducting plane including the intrastack direction. In the case of the sample B, we measured the spectra polarized along the intrastack direction within the conducting plane and along the interlayer direction which is perpendicular to the conducting plane. The spectra polarized in the stacking direction for these two samples were very similar; therefore we have chosen to discuss the spectra measured for the sample A together with the interlayer spectrum of the sample B. Polarized reflectance measurements in the frequency range 600–7000 cm^–1^ were performed using a PerkinElmer 1725 X Fourier-transform spectrometer equipped with an Olympus microscope, a gold grid polarizer, and an Oxford Instruments continuous-flow cryostat. At room temperature, near-infrared (7000–30000 cm^–1^) reflectance spectra were measured using a PerkinElmer Lambda 19 spectrometer, and far-infrared reflectance spectra were probed using a Bruker Equinox 55 FT-IR spectrometer with a IRScope II microscope (7000–15000 cm^–1^), and a Bruker 113 V spectrometer with bolometer detector (50–600 cm^–1^). Raman spectra down to 80 K were recorded using a Micro-Raman Spectrometer (Jobin-Yvon 64000) with 514.5 nm laser light (with 1 cm^–1^ spectral resolution) and a homemade helium cryostat; the laser beam was defocused in the microscope to avoid light-induced deterioration. The optical conductivity σ_1_(ω) was extracted using Kramers–Kronig transformation.^38^ The middle infrared spectra have been extended using 300-K spectra measured in other frequency ranges; ω^–2^ extrapolation has been assumed for the high-frequency data, and a constant applicable for semiconducting materials has been used in the far-infrared range. The decomposition of the complex vibrational bands was performed using standard peak fitting techniques which determine center peak frequency and integral area (intensity). The 10 K optical conductivity spectrum of the sample B polarized in the interlayer direction together with the Raman spectra have already been published in ref (39) and are reproduced here for comprehensive discussion.

## Results

### Crystal Structure

**Figure 1:**
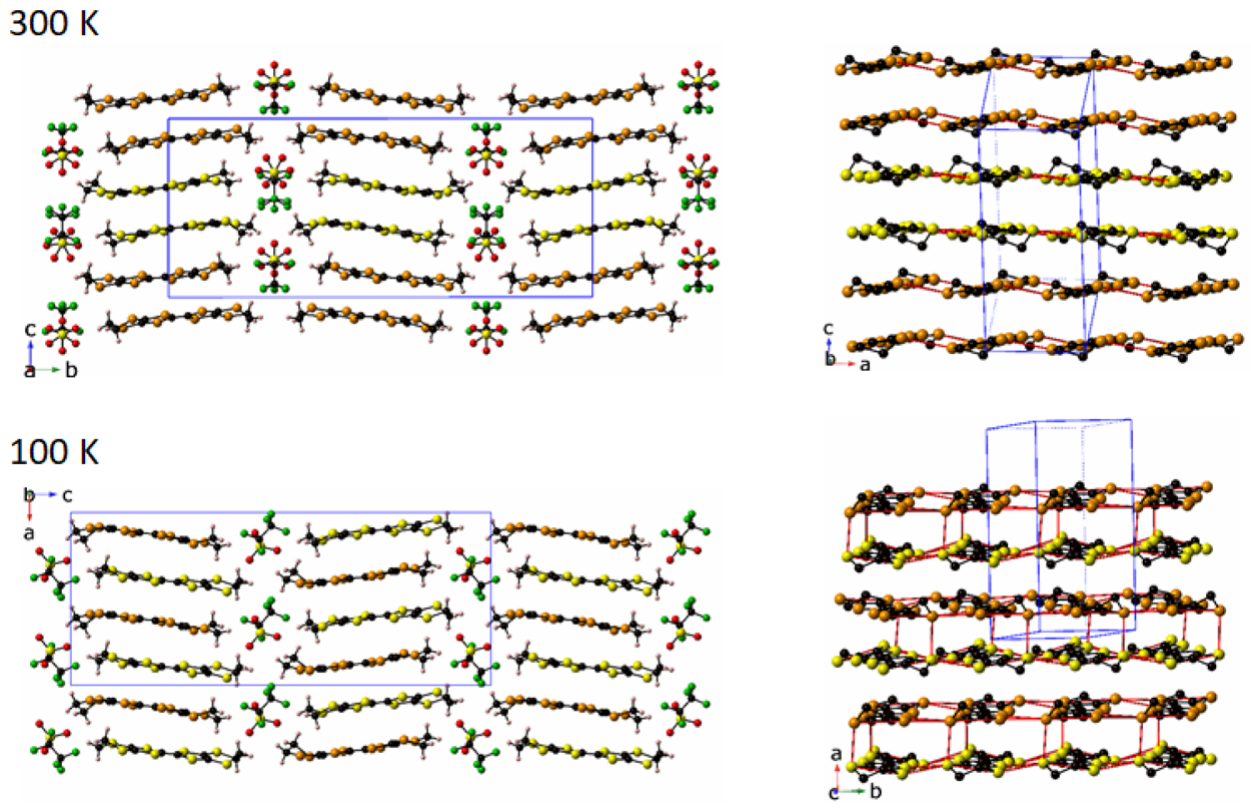
(Left panels) Packing diagram of δ′-(BEDT-TTF)_2_CF_3_CF_2_SO_3_ at 300 K viewed along the *a*-axis, space group *P*2_1_/*m*, and at 100 K, viewed along the *b*-axis, space group *Pca*2_1_. BEDT-TTF molecules A and B are drawn with yellow and orange sulfur atoms, respectively. (Right panels) Packing diagram of BEDT-TTF layer with hydrogen atoms removed for clarity. Red lines indicate intermolecular S···S interactions less than 3.60 Å. The axes used in this figure are those from the original structural data. Note that in the discussion of physical properties of δ′-(BEDT-TTF)_2_CF_3_CF_2_SO_3_ we use the 100 K axes in the whole temperature range in order to avoid misunderstanding; these include the stacking *a*-direction and the interstack *b*-direction within the conducting plane together with the interlayer *c*-direction.

**Figure 2:**
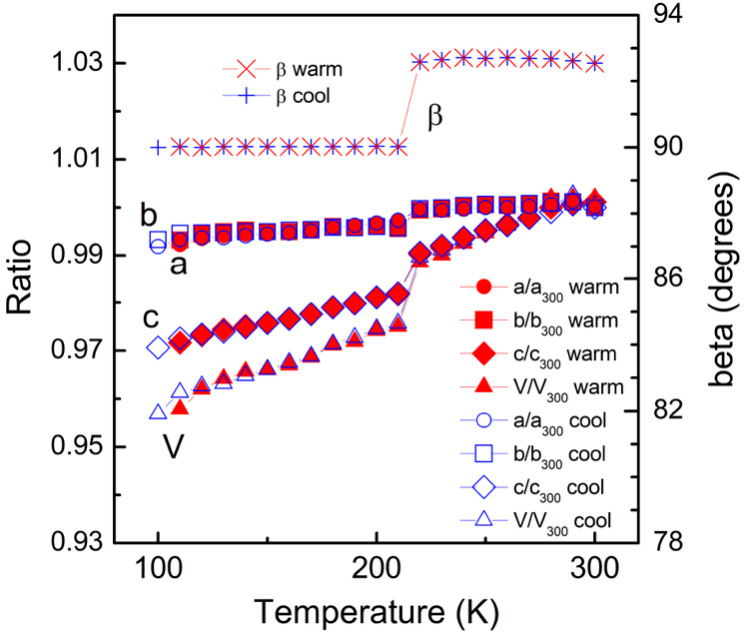
Variable temperature unit cell data of δ′-(BEDT-TTF)_2_CF_3_CF_2_SO_3_ that shows the phase transition. Error bars for the data points are less than, or equal to, the size of the data point symbol.

**Figure 3:**
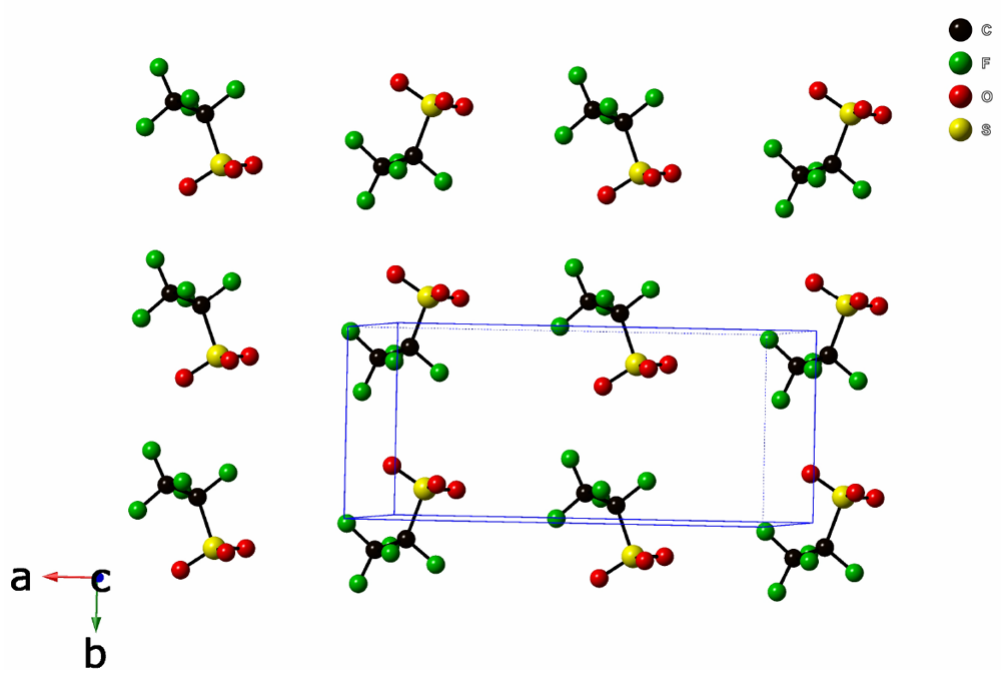
Packing diagram of CF_3_CF_2_SO_3_^–^ anions in δ′-(BEDT-TTF)_2_CF_3_CF_2_SO_3_ at 100 K.

δ′-(BEDT-TTF)_2_CF_3_CF_2_SO_3_ crystallizes in the monoclinic space group *P*2_1_/*m* (Table 1). Its structure is defined by layers of partially oxidized BEDT-TTF molecules separated by anionic layers. The δ′-packing motif is characterized by twisted stacks of dimerized BEDT-TTF radical cations.^7^ All donor layers in this structure are identical. There are two crystallographically nonequivalent BEDT-TTF molecules (hereafter designated as molecules A and B) per unit cell. When the temperature is lowered below about 220 K, δ′-(BEDT-TTF)_2_CF_3_CF_2_SO_3_ undergoes a structural phase transition to the orthorhombic space group *Pca*2_1_, as illustrated in Figure 1. This significant change results in the different principal axes assignment in the high- and low-temperature phases. In the following discussion of physical properties we use the 100 K axes in the whole temperature range in order to avoid misunderstanding. It is interesting that the crystallographically unique molecules change from AABB to ABAB in the transition. The structural modification is clearly seen in the unit cell constants (Figure 2). At room temperature, three of the four crystallographically unique ethylene end-groups of the BEDT-TTF molecules are disordered. As is common in BEDT-TTF salts,^40^ these groups order at temperatures around 100 K. As there are short intermolecular C–H···O and C–H···F contacts between the ethylene groups of the BEDT-TTF molecules and the CF_3_CF_2_SO_3_^–^ anion, we speculate that fluctional disorder in the ethylene groups is correlated with rotational disorder in the CF_3_ and SO_3_ groups of the anion. As the ethylene conformation locks into a single conformation as temperature is lowered, weak hydrogen interactions with the anion’s fluorine and oxygen atoms likely order the configuration of the anion’s end groups. Similar to the case of (BEDT-TTF)_2_SF_5_RSO_3_ (R = CH_2_, CHF, CF_2_) salts,^21^ a number of C–H···O and C–H···F contacts shorter than 2.70 and 2.55 Å, respectively, are observed in δ′-(BEDT-TTF)_2_CF_3_CF_2_SO_3_. Because structural disorder at room temperature results in a lower quality structural determination, a bond length analysis^41^ is less meaningful, but it suggests that the charge on both BEDT-TTF molecules A and B is 0.5(1). However, the high-quality structure determination at 100 K clearly indicates that charge order occurs below the phase transition with a charge of 0.60(2) for molecule A and 0.40(2) for molecule B. Figure 3 displays a packing diagram of CF_3_CF_2_SO_3_^–^ anions at 100 K. These anions form close contacts of the hydrogen-bonding type with the hydrogen atoms of the ethylene end groups of the BEDT-TTF donor molecules.^19^ The details of the crystal structure are provided in the Supporting Information (Figures S1–S6).^42^

### Electronic Structure

**Figure 4:**
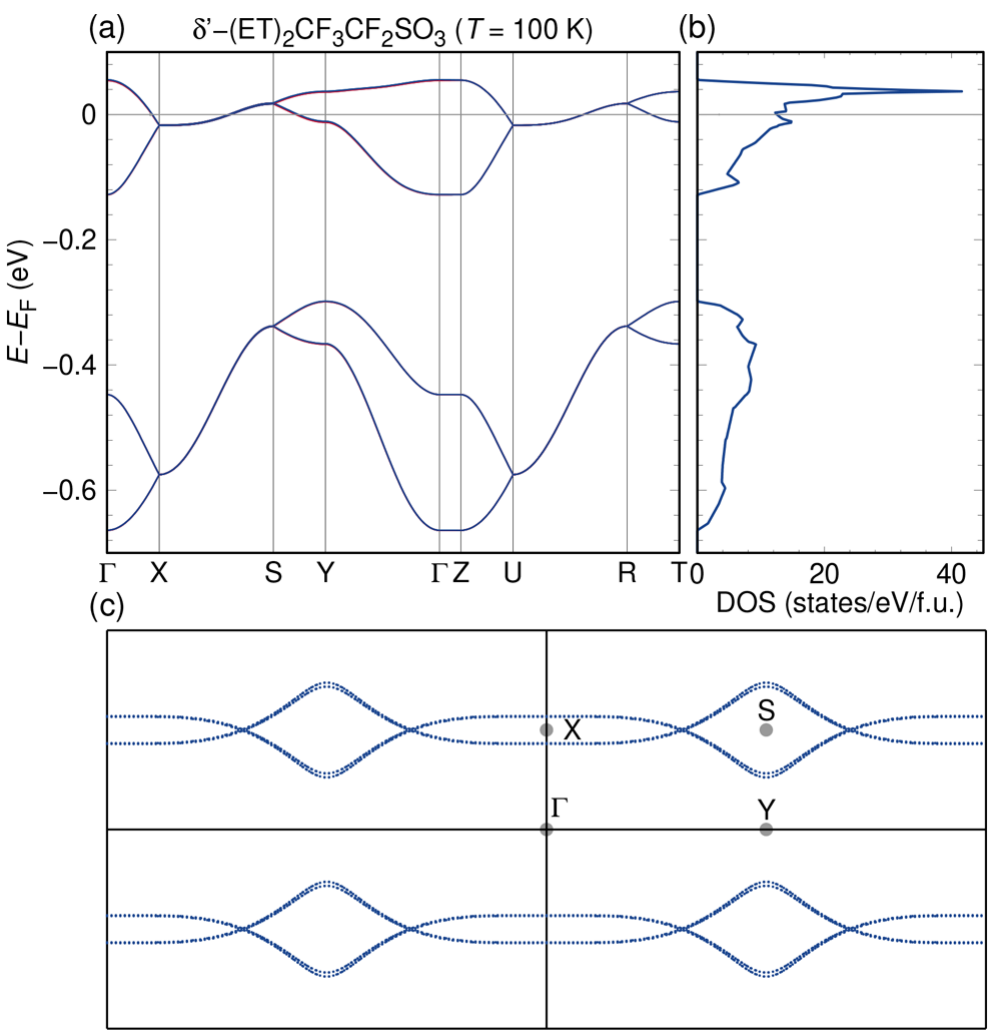
Bandstructure (a), density of states (b), and Fermi surfaces (c) of δ′-(BEDT-TTF)_2_CF_3_CF_2_SO_3_ at *T* = 100 K.

**Figure 5:**
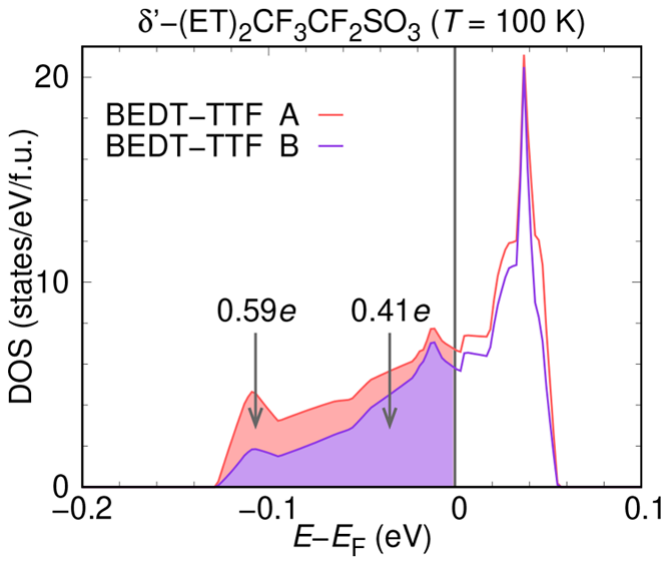
Partial density of states of δ′-(BEDT-TTF)_2_CF_3_CF_2_SO_3_ at *T* = 100 K, separated into contributions from BEDT-TTF molecules A and B. The integrated occupied part yields a charge imbalance of 0.18 electrons between molecule A and molecule B.

The electronic bandstructure, density of states, and Fermi surfaces of δ′-(BEDT-TTF)_2_CF_3_CF_2_SO_3_ at *T* = 100 K are shown in Figure 4. The calculation is performed with four formula units in the *Pca*2_1_ unit cell. The plot shows eight bands arising from the highest occupied molecular orbitals of the BEDT-TTF molecules (Figure 4a); these eight bands are nearly pairwise degenerate, indicating a near perfect two-dimensionality of the system; the splitting due to 3D couplings is only about 2 meV, which shows that the conducting *ab* layers of the material have very little hybridization along *c*. However, closer inspection of the in-plane dispersion reveals that the hopping in the plane is quite anisotropic, making the system almost one-dimensional. In fact, as Figure 4c shows, quasi one-dimensional Fermi surfaces are perpendicular to the stacking *a*-axis. We have investigated the charge order by summing up the densities of states arising from BEDT-TTF molecules A and B separately; this is shown in Figure 5. This integration indicates that at a filling of 0.59*e*, the A molecules have 0.18*e* more charge than the B molecules at a filling of 0.41*e*.

### Transport Measurements

**Figure 6:**
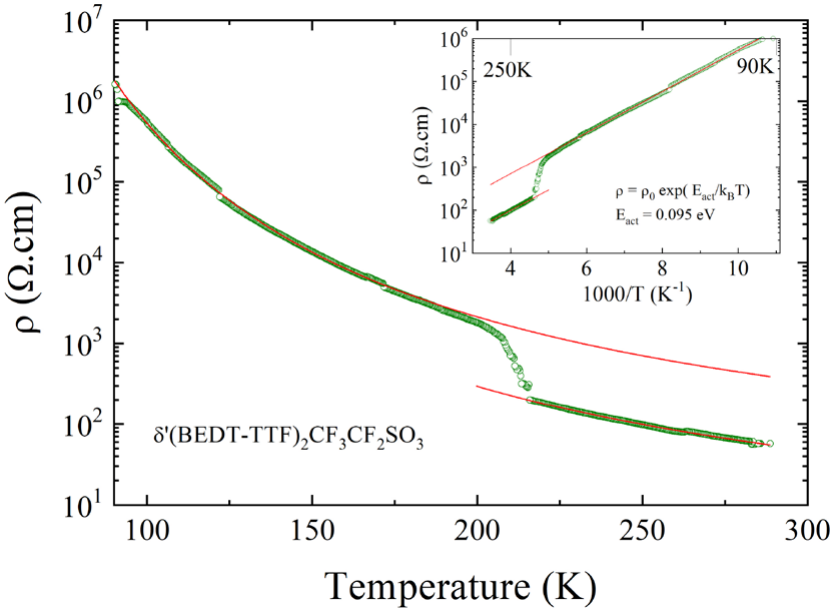
Temperature dependence of the resistivity of δ′-(BEDT-TTF)_2_CF_3_CF_2_SO_3_ measured in the interlayer *c*-direction. The inset shows the Arrhenius plot; the red solid lines are the fit to the data with ρ = ρ_0_ exp(*E*_*act*_/*k*_*B*_*T*) below and above the phase transition.

Figure 6 shows results of resistivity measurements performed in the interlayer *c*-direction on a single crystal of δ′-(BEDT-TTF)_2_CF_3_CF_2_SO_3_. The resistivity shows semiconducting behavior, with a room-temperature conductivity value of ≃0.02 S cm^–1^. The structural phase transition at 220 K is clearly seen as a jump of resistivity due to the change of lattice parameters. The Arrhenius plot of the same data shown as the inset in Figure 6 reveals the activated behavior following the law ρ = ρ_0_ exp(*E*_*act*_/*k*_*B*_*T*) with *E*_*act*_ ≃ 0.095 eV both above and below the phase transition. Therefore, the electrons in δ′-(BEDT-TTF)_2_CF_3_CF_2_SO_3_ are localized with a gap of charge *Δρ* ≃ 2*E*_*act*_ = 0.19 eV. Note that this is a manybody effect which is not captured by the bandstructure calculation (Figure 4). A qualitatively similar semiconducting behavior has been recently observed in the resistivity measurements of the monoclinic δ_*m*_-(BEDT-TTF)_2_TaF_6_ and the orthorhombic δ_*o*_-(BEDT-TTF)_2_TaF_6_ salts, which are both characterized by a phase transition to a charge-ordered phase around room temperature.^14^

### Optical Response of the Insulating State

**Figure 7:**
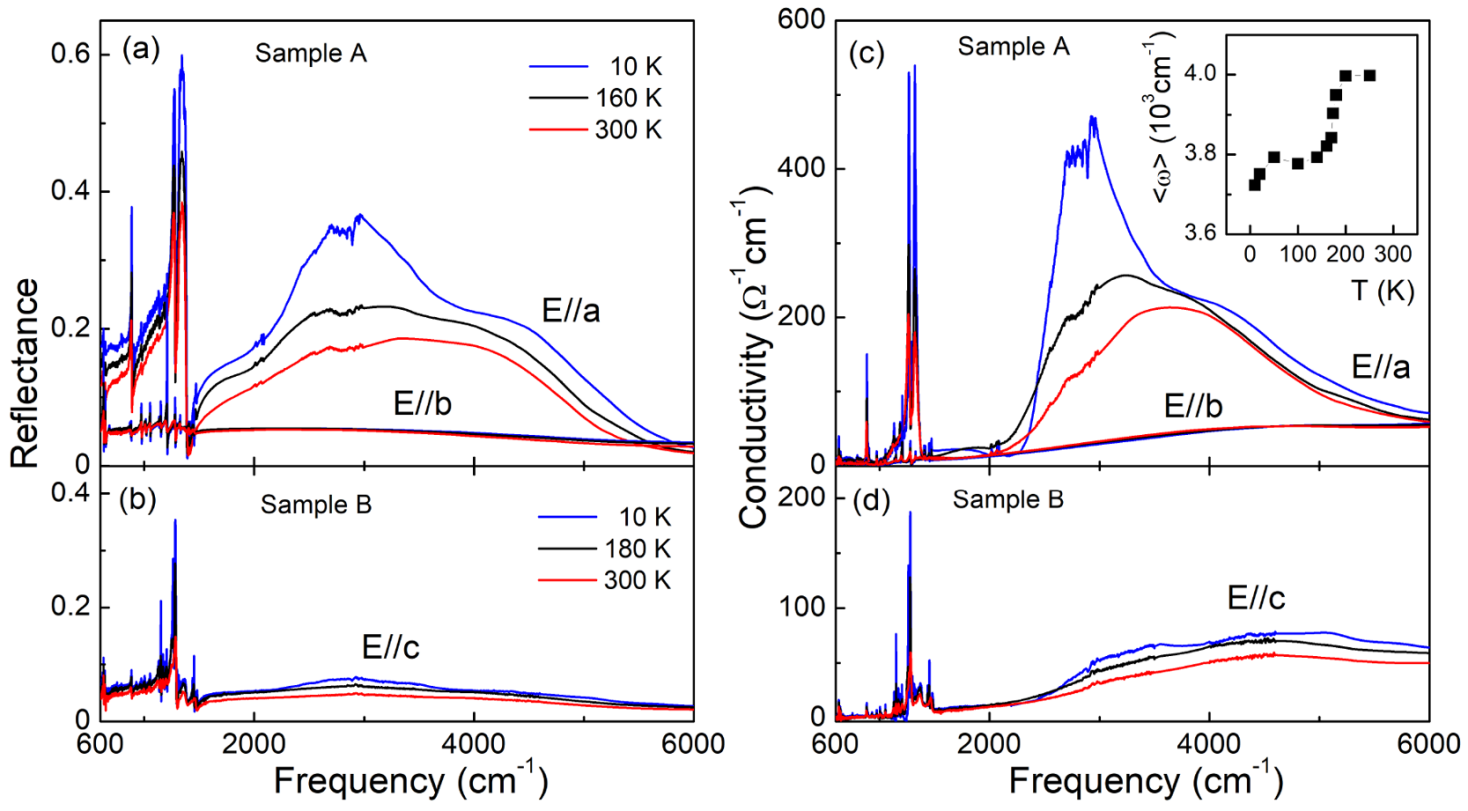
Polarized reflectance (a, b) and optical conductivity spectra (c, d) of δ′-(BEDT-TTF)_2_CF_3_CF_2_SO_3_ measured for samples A ((a, c) −*E*∥*a*, *E*∥ *b*), and B ((b, d) −*E*∥*c*), for selected temperatures between 300 and 10 K. The inset in panel c shows the temperature dependence of the center of spectral weight in the stack *a* direction.

Figure 7 displays the infrared reflectance and optical conductivity spectra of δ′-(BEDT-TTF)_2_CF_3_CF_2_SO_3_ at selected temperatures, polarized in the three principle polarization directions, the stack *a*- and interstack *b*-directions within the conducting plane, and interlayer *c*-direction. In the δ-type structure, twisted dimers promote strong intradimer interaction. In fact, significantly higher reflectance is detected in the direction parallel to the stacks of BEDT-TTF dimers within the conducting BEDT-TTF plane (*E⃗* ∥*a*) in agreement with the band structure calculations, with the reflectance value below 0.5 (Figure 7a). Lower reflectance levels are observed along *b*- and *c*-directions (Figure 7, parts a and b), reproducing the quasi-one-dimensional nature of the electronic structure. A characteristic feature of the optical conductivity spectra calculated using the Kramers–Kronig analysis of the measured reflectance is the broad mid-infrared electronic band between 2000 and 6000 cm^–1^ in the stack direction accompanied by strong molecular vibrational modes below 1500 cm^–1^ (Figure 7c). Most of these vibrational features originate from the electron–molecular vibration (*e–mv*) coupling of the totally symmetrical modes of the BEDT-TTF molecule with the aforementioned low-lying electronic transition within the dimerized structure.^43,44^ At the same time, a weak electronic response together with normally IR-active vibrational modes is found in the interstack *b*- and interlayer *c*-directions (Figure 7, parts c and d).

The main effect of a temperature decrease from 300 to 10 K is the optical conductivity increase in the stack direction around 2900 cm^–1^ in the range of the electronic transitions. While at high temperatures we detect a single slightly asymmetric band centered at about 3700 cm^–1^, a clear doublet structure is evident at low temperature with two band components centered at ≈2900 and ≈3800 cm^–1^ at 10 K.^45^ There is no change in the overall character of the optical spectra of δ′-(BEDT-TTF)_2_CF_3_CF_2_SO_3_ when lowering the temperature through the structural phase transition at 220 K, in agreement with the results of resistivity measurements. Relatively strong temperature dependence in the narrow temperature range below 220 K in the vicinity of 2900 cm^–1^ is reflected in the downshift of the center of spectral weight in the stack direction, defined here as ⟨ω⟩ ≡ ∫_2100_^6000^σ(ω) *ω* d*ω*/∫_2100_^6000^ σ(ω) d*ω*, by about 230 cm^–1^ when lowering the temperature through the phase transition (inset in Figure 7c). At the same time the low-frequency edge of the electronic band allows the estimation of the optical gap of about 2300 cm^–1^ at 10 K, a value higher than ≈1520 cm^–1^ estimated based on the resistivity measurements. A similar anisotropic optical response of the conducting layer has been detected in the β′-(BEDT-TTF)_2_CF_3_CF_2_SO_3_ dimer–Mott insulator, the material obtained as a minority phase in the same synthesis and recently suggested to undergo a charge order transition below 25 K.^26^

**Figure 8:**
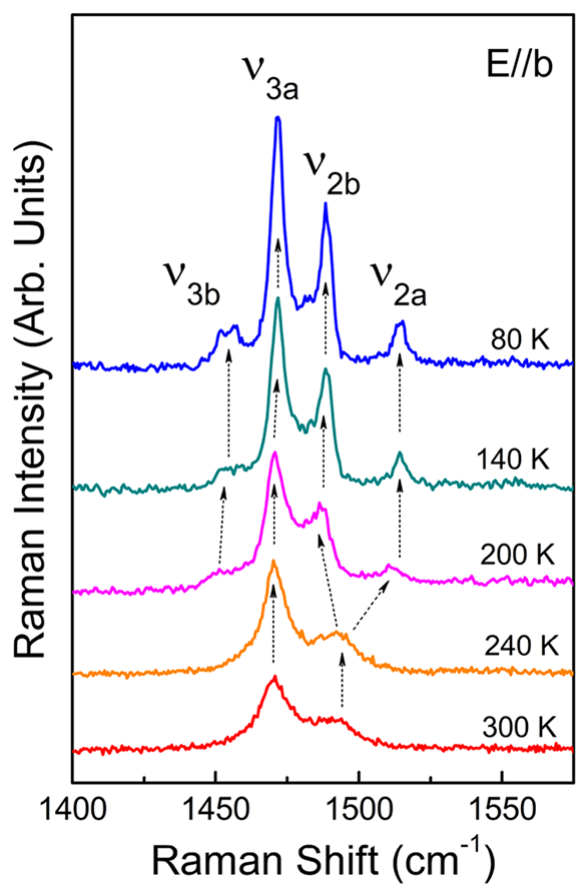
Raman spectra of δ′-(BEDT-TTF)_2_CF_3_CF_2_SO_3_ at selected temperatures in the frequency range of the charge-sensitive BEDT-TTF modes ν_2_ and ν_3_ that split at 200 K into components ν_2a_, ν_2b_ and ν_3a_, ν_3b_. Note that the material remains in the CO state at low temperature.

**Figure 9:**
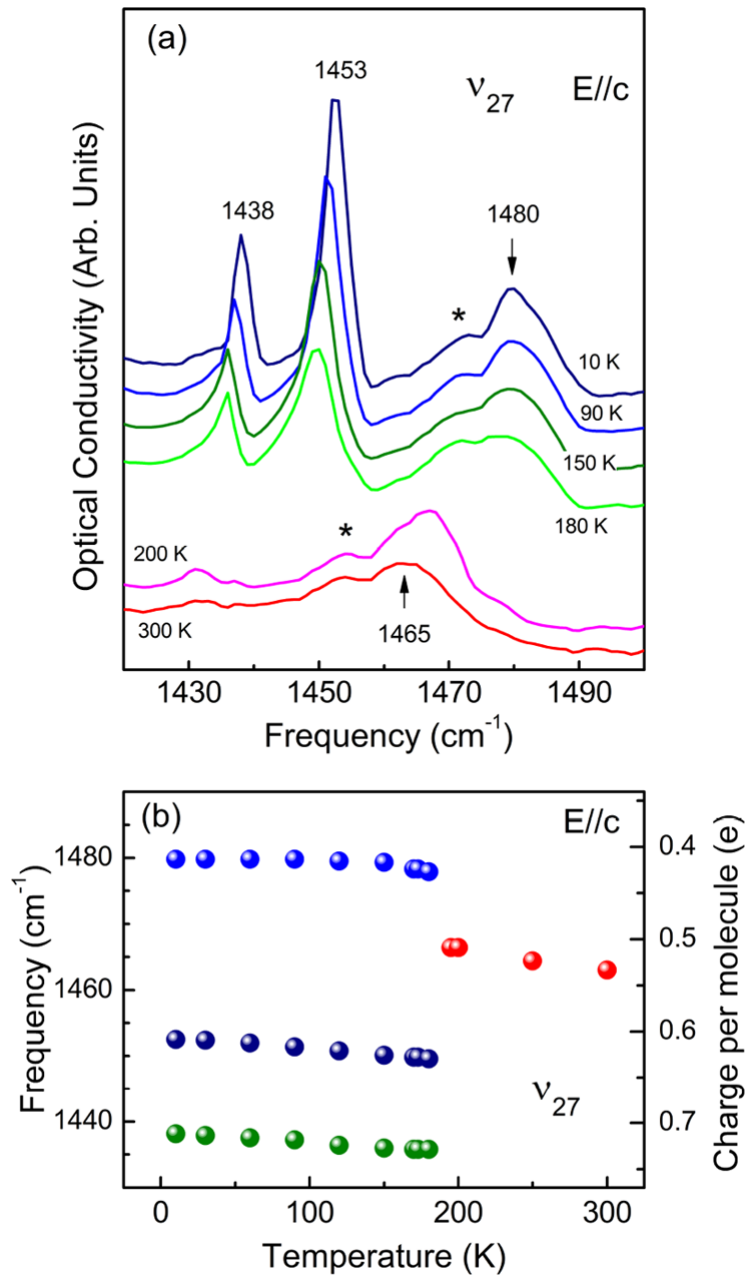
(a) Optical conductivity spectra of δ′-(BEDT-TTF)_2_CF_3_CF_2_SO_3_ polarized in the interlayer *c*-direction, in the frequency range of the charge-sensitive vibrational ν_27_ mode centered at ≈1465 cm^–1^ at room temperature that shows splitting in the charge-ordered phase below 200 K into three components, 1438, 1453, and 1480 cm^–1^. Another possible ν_27_ mode component is marked with the asterisk; the spectra are shifted for clarity. (b) Temperature dependence of the ν_27_ mode frequencies. The scale on the right correlates the frequency to the charge per molecule calculated with the formula from ref (53). Error bars for the data points are less than, or equal to, the size of the data point symbol.

**Figure 10:**
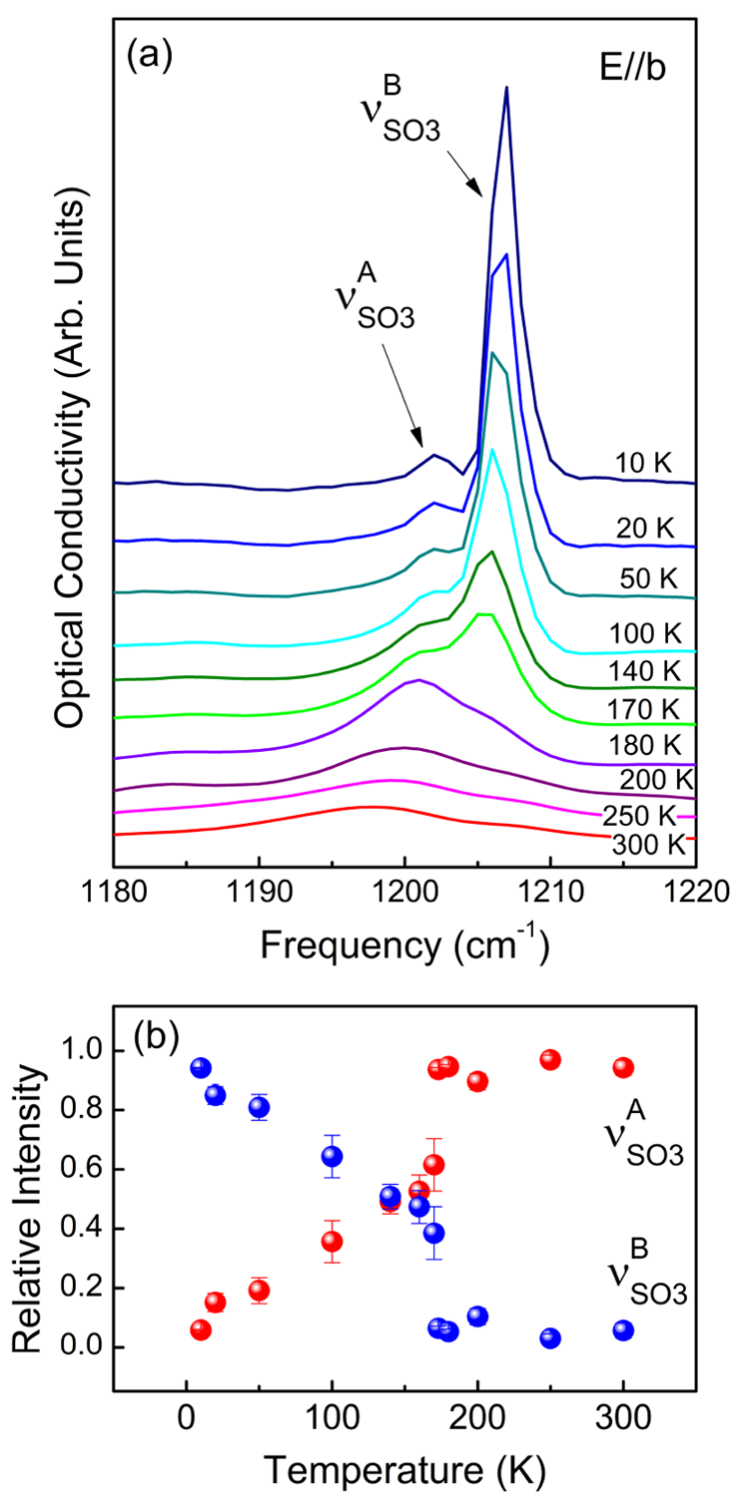
(a) Optical conductivity spectra of δ′-(BEDT-TTF)_2_CF_3_CF_2_SO_3_ at selected temperatures in the frequency range of the anion SO_3_ stretching mode composed of two components, ν_SO_3__^A^ and ν_SO_3__^B^; the spectra are shifted for clarity. (b) Temperature dependence of the relative intensity of the two SO_3_ components calculated with respect to the total intensity of the doublet structure. Error bars estimated statistically are also shown.

The optical response of a dimer–Mott insulator discussed within half-filling is characterized by two mid-infrared electronic absorption bands, the Hubbard band attributed to the interdimer charge transfer, and the dimer band related to the intradimer charge transfer.^2,46−49^ Intensity of the Hubbard band usually strongly depends on temperature, therefore we assign the low-temperature 2900 cm^–1^ component in the optical spectra of δ′-(BEDT-TTF)_2_CF_3_CF_2_SO_3_ polarized in the stack direction as the Hubbard band, and the 3800 cm^–1^ component as the dimer band. It is known that the position of the Hubbard band allows estimation of the effective Coulomb interaction *U*_eff_ and its half width at half-maximum is proportional to the electronic bandwidth *W*/ℏ that is related to the kinetic energy of the electrons; these two values can be used for estimating the relative size of Coulomb correlations *U*_eff_/*W*.^50^ The 2900 cm^–1^ band is rather narrow, with the bandwidth of about 400 cm^–1^ at 10 K, and is centered at a slightly higher frequency than in case of the model dimer–Mott κ-phase BEDT-TTF salts.^46−48,50^ This results in *U*_eff_/*W* ≈ 7, the value that confirms strong Coulomb correlations, in line with the insulating behavior of δ′-(BEDT-TTF)_2_CF_3_CF_2_SO_3_ in the whole temperature range. Modification of the optical spectra below 220 K is accompanied by splitting of the charge-sensitive vibrational modes both in Raman and infrared spectra, as discussed in detail in the next section (Figures 8 and 9). Other vibrational features display splitting related with both the structural modification and charge order (Figures 10 and S8 (Supporting Information)^42^).

### Charge-Ordered State

In order to give detailed information on the charge-ordered states suggested by the crystal structure measurements, we focus on the normal modes of the BEDT-TTF molecule involving C=C stretching vibrations sensitive to charge and therefore widely used in investigations of the local charge distribution.^51−53^ Assuming the planar *D*_2*h*_ molecular symmetry, these modes are labeled ν_2_(A_g_), assigned mainly to symmetric ring C=C stretching, ν_3_(A_g_) to bridge C=C stretching, and ν_27_(B_1u_) to antisymmetric ring C=C stretching.

Figure 8 reports the Raman spectra of δ′-(BEDT-TTF)_2_CF_3_CF_2_SO_3_ at several selected temperatures, in the spectral range of the Raman-active totally symmetric ν_2_ and ν_3_ modes. At room temperature, one can find two broadened bands at 1471 and 1494 cm^–1^. When the temperature is lowered below about 200 K, the splitting is observed, and the Raman spectra display as much as four peaks, gradually narrowing with further temperature decrease. The band at 1471 cm^–1^ remains relatively unaltered starting from 300 K down to the lowest temperature, therefore it is assigned as the ν_3_ component usually discussed as the in-phase combination of the ν_3_(A_g_) modes in a centrosymmetric dimer, not sensitive to charge (ν_3a_ in Figure 8).^53^ The band that emerges at 1455 cm^–1^ is attributed to another ν_3_ component activated in a modified environment below the structural phase transition (ν_3b_). The remaining modes are identified as the charge-sensitive ν_2_ components. According to the formula^53^
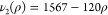
1where charge ρ is given in units of *e*, the observed frequency of the single ν_2_ mode centered at 1494 cm^–1^ at room temperature indicates the presence of BEDT-TTF molecules carrying average charge +0.5*e*. Accordingly, the two nonequivalent BEDT-TTF molecules in the unit cell are most probably almost equally charged. Below the 200 K phase transition, the band clearly splits into two features centered at 1489 (ν_2b_) and 1514 cm^–1^ (ν_2a_) at 80 K, representing BEDT-TTF molecules with average charge +0.66*e*, and +0.44*e* respectively. Thus, the Raman spectra confirm that the charge-ordered state is established in δ′-(BEDT-TTF)_2_CF_3_CF_2_SO_3_ below 200 K, with the charge disproportionation ≈0.2*e*.

In the vibrational range of the conductivity spectra, we focus on the infrared-active *ungerade ν*_27_(B_1u_) mode that is usually observed in the spectra polarized in the direction parallel to the BEDT-TTF long molecular axis and therefore observed in δ′-(BEDT-TTF)_2_CF_3_CF_2_SO_3_ in the interlayer *E⃗*∥*c* direction (Figure 9a). The ν_27_ mode not perturbed by *e–mv* coupling^52^ is regarded as the best probe to estimate the local charge because of the strongest dependence on charge among the BEDT-TTF modes.^53^ At room temperature, ν_27_ is observed at ≈1465 cm^–1^ as a rather broad feature with a shoulder (asterisk in Figure 9a) that suggests a doublet structure. The mode is relatively narrow comparing results reported for organic superconductor β″-(BEDT-TTF)_2_SF_5_CH_2_CF_2_SO_3_ and metal β″-(BEDT-TTF)_2_SF_5_CHFSO_3_ that have been discussed in terms of charge fluctuations,^51^ in agreement with insulating properties. On lowering the temperature, the ν_27_ mode is becoming more pronounced until sharp splitting into three well-defined components at 1438, 1453, and 1480 cm^–1^ on entering the low-temperature phase that can be therefore identified as charge-ordered. Interestingly, the unique shape of ν_27_ above the phase transition is closely retained below but shifted to about 1480 cm^–1^. This strongly suggests that the doublet structure observed above 200 K is related with the presence of two crystallographically independent but nearly equivalent molecules in the unit cell, otherwise equally charged, as suggested by the presence of the single ν_2_ band in the Raman spectra. Taking into account the three low-temperature modes and the shoulder, we observe as much as four distinct ν_27_ components in the charge-ordered phase, instead of the two expected based on our crystal structure measurements. This implies that the actual symmetry below the structural phase transition is lower than expected, at least from the point of view of the scale of the infrared experiment. Similar multiple splitting of the charge-sensitive ν_27_ mode has been recently detected in the insulating phase of β″-(BEDT-TTF)_2_Hg(SCN)_2_Cl.^54^

In order to evaluate charge disproportionation in δ′-(BEDT-TTF)_2_CF_3_CF_2_SO_3_ based on ν_27_, we apply the linear relationship between the frequency and charge ρ^53^
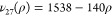
2to the frequencies that were obtained in the fitting procedure of the three ν_27_ mode components using Lorentzian functions. Figure 9b reports the temperature dependence of the ν_27_ frequencies together with the corresponding charge per molecule. The frequency of the single ν_27_ component above 200 K is consistent with ρ = 0.5*e*, in agreement with the crystal structure and Raman experiment. On the other hand, splitting at low temperature into multiple components gives rise to a question which of them are actually related to charge difference because they all appear as a result of unfolding degeneracy by both charge ordering and the significant structural modification. Here we argue that the two low-temperature components at 1453 and 1480 cm^–1^ located in the vicinity of the high-temperature 1465 cm^–1^ band are mostly affected by the charge-ordering transition. Therefore, we evaluate the charge disproportionation using the fractional charges 0.61 and 0.41*e* calculated for the 1453 and 1480 cm^–1^ ν_27_ components, respectively, as ≈0.2*e*, which is in perfect agreement with both the calculation based on the Raman-active ν_2_ mode and the band structure calculations as well as the respective estimation based on the crystal structure. The charge disproportionation in δ′-(BEDT-TTF)_2_CF_3_CF_2_SO_3_ is basically temperature independent in the charge-ordered phase (Figure 9b). The value ≈0.2*e* can be compared with similar charge differences observed for β″-(BEDT-TTF)_2_SF_5_CH_2_CF_2_SO_3_,^51^ β″-(BEDT-TTF)_2_Hg(SCN)_2_Cl,^54^ and κ″-(BEDT-TTF)_2_Hg(SCN)_2_Cl.^55^ Surprisingly, it is significantly smaller than the about 0.4–0.7*e* observed in less conducting δ-phase (BEDT-TTF)_2_MF_6_ salts, where M = P, As, Sb, and Ta.^14,16,17^

The charge order phase transition in δ′-(BEDT-TTF)_2_CF_3_CF_2_SO_3_ is related to significant structural change that influences hydrogen bonding type interactions between conducting BEDT-TTF and anion layers. In particular, there exist close contacts involving the SO_3_ group of the CF_3_CF_2_SO_3_^–^ anion and ethylene groups of BEDT-TTF. We now focus on the stretching SO_3_ mode that is observed in the infrared spectra near 1200 cm^–1^ (vibrational properties of CF_3_CF_2_SO_3_^–^ are provided in Figure S7 and Table S1 in the Supporting Imformation).^42^ In the optical conductivity spectra of δ′-(BEDT-TTF)_2_CF_3_CF_2_SO_3_ polarized in the interstack *b*-direction, we can easily identify a doublet structure that displays significant temperature dependence (Figure 10a). The line width of the mode becomes significantly smaller below the 200 K structural phase transition, which indicates some ordering most probably concerning both the ethylene end groups of BEDT-TTF and the anion layer. In order to quantify modifications of the two mode components we have fitted the SO_3_ band using two spectral functions. Figure 10b presents the relative intensities of ν_SO_3__^A^ and ν_SO_3__^B^ with respect to the total intensity of the doublet structure as a function of temperature. While above the phase transition most of the intensity is localized in the lower frequency ν_SO_3__^A^ component centered at about 1202 cm^–1^, below the phase transition the intensity is shifted to the ν_SO_3__^B^ component at ≈1207 cm^–1^. Thus, our observation confirms that the structural change at 200 K is related to a significant modification of the interaction between the anion and donor layers.

## Discussion

The charge order pattern in the dimerized systems strongly depends on the intersite Coulomb repulsion *V* that can be approximated by the inverse of distance between neighboring molecular centers, a value usually more uniform in the conducting layer than transfer integrals.^56^ Thus, a variety of stripe and nonstripe patterns can be realized, depending on the specific structure of the conducting layer minimizing the energy of the system. The detailed information on the charge order state can be extracted from both the structural studies, electronic band structure calculations, and optical measurements.

We now focus on the electronic part of the optical conductivity spectra of δ′-(BEDT-TTF)_2_CF_3_CF_2_SO_3_ that contains information about correlations. While in the half-filled Hubbard picture the frequency of the Hubbard band is related to *U*, less dimerized quarter-filled systems with significant intersite Coulomb repulsion *V* and CO states give rise to a more complicated picture with the Hubbard band positions depending on both *U* and *V*.^18,57^ In such a scenario, the shape of the mid-infrared electronic band is closely related to the charge pattern. Along the stripe axis, the excitations arise between charge-rich molecules only and can be well described as half-filled Mott insulators with the Hubbard band shifted to *U* – *V*_*i*_ (where *V*_*i*_ is the intersite Coulomb repulsion along the *i* axis). Then, the electrodynamic response along a direction with alternating charge-poor and charge-rich sites is characterized by an asymmetric broadened band centered at the *V*_*i*_ frequency, significantly lower than in the stripe case. The respective mid-infrared excitation in δ′-(BEDT-TTF)_2_CF_3_CF_2_SO_3_ (Figure 7c) is identified as the dimer–Mott response which would suggest a stripe pattern along the stacking direction. On the other hand, the band acquires an asymmetric shape at low temperature pointing to the 1010-type pattern. In fact, with the modest charge disproportionation ≈0.2*e* we should probably expect a mixed response because each transition involves both on-site *U* and intersite *V*_*i*_ Coulomb repulsions.^57^ Therefore, the shape of the mid-infrared electronic transition is not conclusive from the point of view of the CO pattern. Yet, there is a hint related with a characteristic vibrational structure that appears as a dip on top of the band at ≈2900 cm^–1^ (Figure 7c). This feature is attributed to the overtone of the ν_3_ mode strongly coupled with the electronic transition, which is known to be activated due to the anharmonicity of the energy potential in the presence of charge disproportionation within a BEDT-TTF dimer.^58^ Therefore, we consider a charge order pattern with charge disproportionation within a dimer.

**Figure 11:**
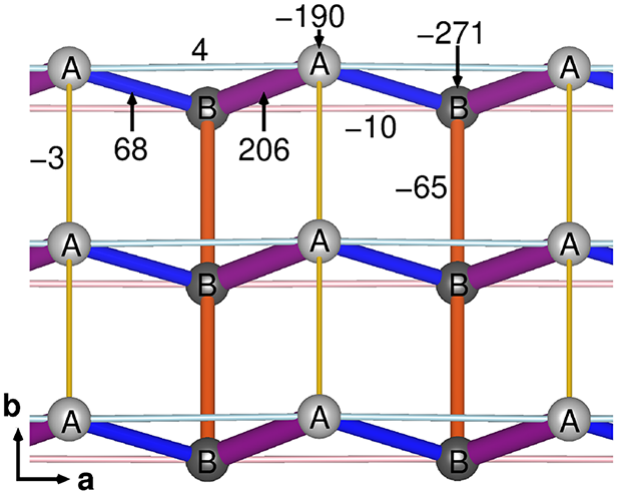
Charge order pattern in δ′-(BEDT-TTF)_2_CF_3_CF_2_SO_3_ including the onsite energies and hopping parameters in meV as extracted from the tight binding Hamiltonian for the 100 K structure. Here, light circles represent BEDT-TTF A molecules with more charge (0.59*e*) compared to the B molecules (0.41*e*) marked with dark circles. Note that *a* is the stack direction and *b* is the interstack direction within the conducting *ab*-plane.

Here we propose based on both the crystal structure and the electronic structure calculations that a CO pattern with the horizontal stripe along interstack *b*-direction is realized in δ′-(BEDT-TTF)_2_CF_3_CF_2_SO_3_ (see Figure 11). Note that the strongest tight binding parameter (*t* = 206 meV) connects A molecules with B molecules along the *a* stacking direction, forming BEDT-TTF dimers.

It is known that the stripe pattern can be related to the enhanced one-dimensional coupling of neighboring spins on the charge-rich sites,^57^ in agreement with the spin susceptibility value decreasing with lowering the temperature.^19^ Such a charge order pattern is rather unique among dimer–Mott insulators based on BEDT-TTF. Recently, a checkerboard CO pattern has been suggested for the δ-phase (BEDT-TTF)_2_TaF_6_ salts,^14^ similar to β-(*meso*-DMBEDT-TTF)_2_PF_6_.^59^

## Conclusions

The δ′-(BEDT-TTF)_2_CF_3_CF_2_SO_3_ organic conductor has been synthesized and characterized using X-ray diffraction, resistivity, and optical property measurements, together with band-structure calculations. This layered material is characterized by the dimerized structure of the conducting layer, the presence of the hydrogen bonding-type interactions between the CF_3_CF_2_SO_3_^–^ anion and ethylene groups of the BEDT-TTF donor molecule and the optical response characteristic for a dimer–Mott insulator. δ′-(BEDT-TTF)_2_CF_3_CF_2_SO_3_ undergoes around 200 K a structural transition to a low-temperature phase, as evidenced by the thermal variation of structural parameters and resistivity in the interlayer direction, which is accompanied by charge ordering as evidenced by the splitting of the charge-sensitive ν_2_ and ν_27_ vibrational BEDT-TTF modes. The interaction between the anion and donor layers is modified by the structural change at 200 K, as revealed by the behavior of the SO_3_ stretching modes of CF_3_CF_2_SO_3_^–^. Our results strongly suggest that the horizontal stripe charge order pattern with the charge disproportionation of the order of 0.2*e* within a dimer is established below 200 K. Note that this agrees very well with our electronic structure calculation for the 100 K structure.

## Supporting Information Available

Additional crystal structure information including thermal ellipsoid plots, infrared spectra of the CF_3_CF_2_SO_3_^–^ anion, and selected vibrational features characterized by temperature-dependent splitting (PDF)
