# Supplementary material for: Charge-Ordering and Structural Transition in the New Organic Conductor δ′-(BEDT-TTF)2CF3CF2SO3
Source: J Phys Chem C Nanomater Interfaces. 2022 Jan 25;126(4):1890–900. doi: 10.1021/acs.jpcc.1c09458 (PMC8819691; doi:10.1021/acs.jpcc.1c09458)
Supplement: Supplementary file 1 — jp1c09458_si_001.pdf [file jp1c09458_si_001.pdf]

Supporting Information for:

Charge Ordering and Structural Transition in the New Organic Conductor δ'-(BEDT-TTF)$_2$CF$_3$CF$_2$SO$_3$

Iwona Olejniczak[1], Bolesław Barszcz[1], Pascale Auban-Senzier[2], Harald O. Jeschke[3], Roman Wojciechowski[4], and John A. Schlueter[5,6]

[1]Institute of Molecular Physics, Polish Academy of Sciences, Smoluchowskiego 17, 60-179 Poznań, Poland
[2]Laboratoire de Physique des Solides, Université Paris-Saclay, UMR 8502 CNRS, Université Paris-Sud, Orsay 91405, France
[3]Research Institute for Interdisciplinary Science, Okayama University, Okayama 700-8530, Japan
[4]Department of Molecular Physics, Faculty of Chemistry, Technical University of Łódź, Żeromskiego 116, 90-924 Łódź, Poland
[5]Materials Science Division, Argonne National Laboratory, Argonne, IL 60439, USA
[6]Division of Materials Research, National Science Foundation, 2415 Eisenhower Avenue, Alexandria, VA 22314, USA

SI1 Crystal structure of δ'-(BEDT-TTF)$_2$CF$_3$CF$_2$SO$_3$

δ'-(BEDT-TTF)$_2$CF$_3$CF$_2$SO$_3$, where BEDT-TTF is bis(ethylenedithio)tetrathiafulvalene, belongs to the family (BEDT-TTF)$_2$RR′SO$_3$, where R = SF$_5$, CF$_3$, and R′ = CH$_2$, CF$_2$, CHF, CHFCF$_2$, CH$_2$CF$_2$, of entirely organic conductors including a highly tunable anion.[1, 2] The anions form close contacts of the hydrogen-bonding type with the hydrogen atoms of the ending ethylene groups of the BEDT-TTF donor molecules [3] and thus strongly influence electronic properties of the material.

δ'-(BEDT-TTF)$_2$CF$_3$CF$_2$SO$_3$ crystallizes in the monoclinic space group P2$_1$/m. Its structure is characterized by layers of partially oxidized BEDT-TTF molecules separated by anionic layers. There are two crystallographically nonequivalent BEDT-TTF molecules, designated as molecules A and B, per unit cell. At 100 K, there is no significant disorder in the cation layers with the ethylene groups of both molecules A and B in an eclipsed conformation (Figures S1, S2). Analysis of the bond lengths, normalized for a charge of -1 on the CF$_3$CF$_2$SO$_3^-$ anion, yields an oxidation state of +0.60 for molecule A, and +0.40 for molecule B.[4] At room temperature, structural disorder is present both in cation and anion layers (Figures S4-S6).

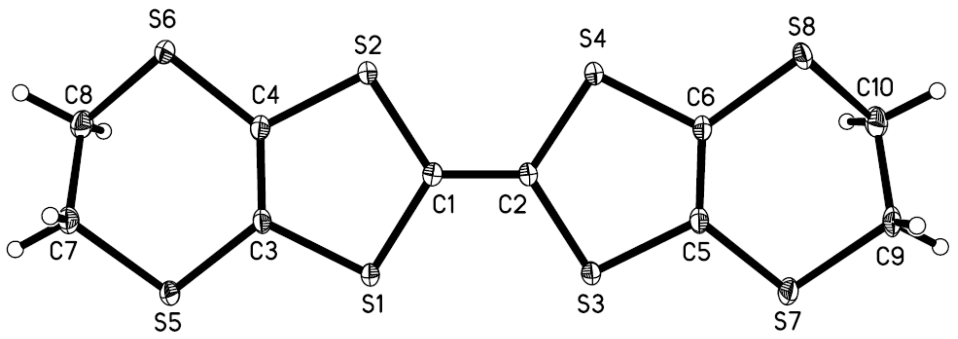

Figure S1. Thermal ellipsoid plot (50% probability level) with atom labeling scheme for the BEDT-TTF molecule (A) in the δ'-(BEDT-TTF)$_2$CF$_3$CF$_2$SO$_3$ structure at 100 K. Hydrogen atoms are drawn as spheres with arbitrary radius. Analysis of the bond lengths, normalized for a charge of -1 on the CF$_3$CF$_2$SO$_3^-$ anion, yields an oxidation state of +0.60. The ethylene groups are ordered in an eclipsed conformation.

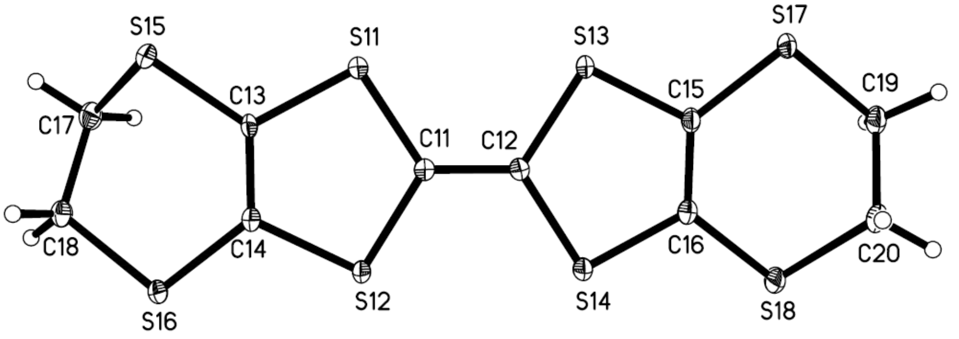

Figure S2. Thermal ellipsoid plot (50% probability level) with atom labeling scheme for the BEDT-TTF molecule (B) in the δ′-(BEDT-TTF)$_2$CF$_3$CF$_2$SO$_3$ structure at 100 K. Hydrogen atoms are drawn as spheres with arbitrary radius. Analysis of the bond lengths, normalized for a charge of -1 on the $CF_3CF_2SO_3^-$ anion, yields an oxidation state of +0.40. The ethylene groups are ordered in an eclipsed conformation.

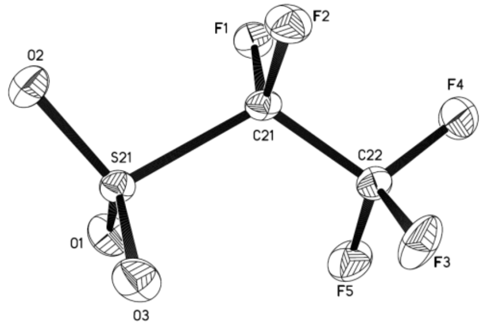

Figure S3. Thermal ellipsoid plot (50% probability level) with atom labeling scheme for the $CF_3CF_2SO_3^-$ anion in the δ′-(BEDT-TTF)$_2$CF$_3$CF$_2$SO$_3$ structure at 100 K.

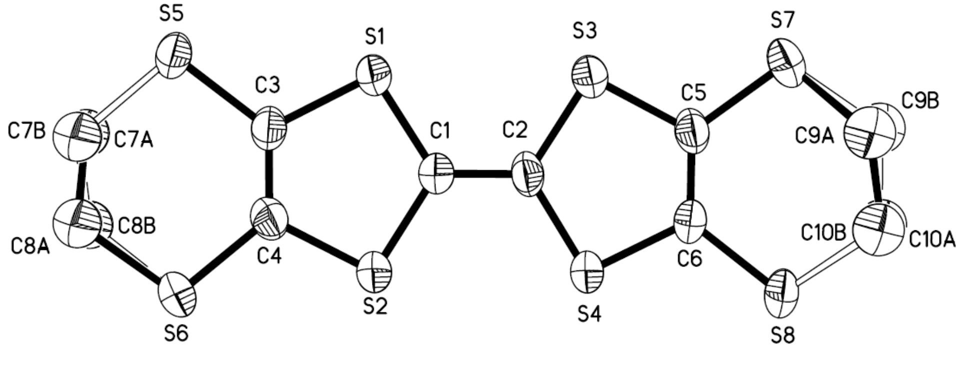

Figure S4. Thermal ellipsoid plot (50% probability level) with atom labeling scheme for the BEDT-TTF molecule (A) in the δ′-(BEDT-TTF)$_2$CF$_3$CF$_2$SO$_3$ structure at 300 K. Hydrogen atoms are drawn as spheres with arbitrary radius. The significant uncertainty in bond lengths due to structural disorder renders it impossible to make an accurate determination of the charge state.

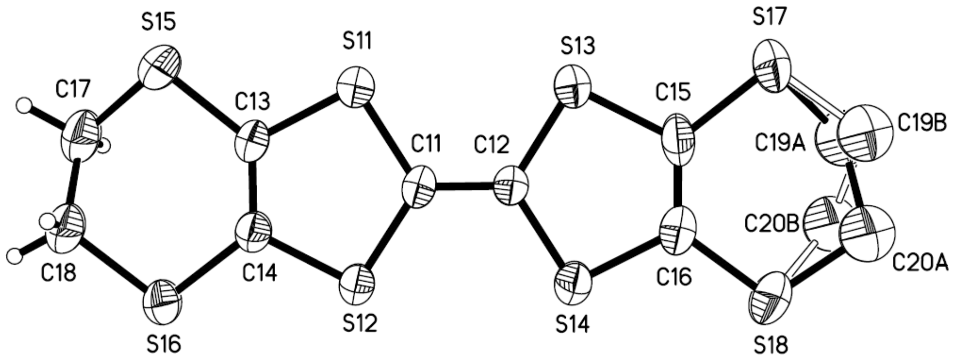

Figure S5. Thermal ellipsoid plot (50% probability level) with atom labeling scheme for the BEDT-TTF molecule (B) in the δ′-(BEDT-TTF)$_2$CF$_3$CF$_3$SO$_3$ structure at 300 K. Hydrogen atoms are drawn as spheres with arbitrary radius. One of the ethylene groups is disordered over two (eclipsed/staggered) conformations with an approximately equal distribution. The significant uncertainty in bond lengths due to structural disorder renders it impossible to make an accurate determination of the charge state.

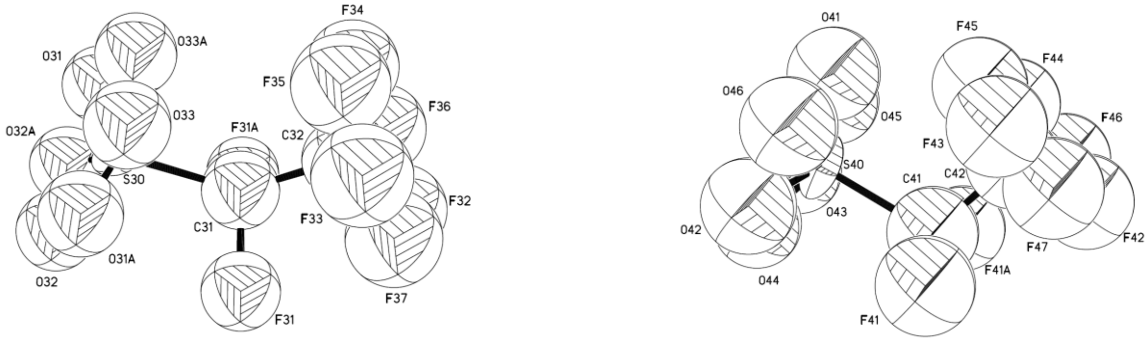

Figure S6. The two crystallographically independent CF$_3$CF$_3$SO$_3^-$ anions in the δ′-(BEDT-TTF)$_2$CF$_3$CF$_3$SO$_3$ structure at 300 K, including the atom labeling scheme. Thermal ellipsoids are drawn at the 50% probability level. Considerable disorder exists in both the CF3 and SO3 groups.

SI2 Infrared-active vibrational modes of the CF$_3$CF$_2$SO$_3^-$ anion: experiment and DFT calculations

In order to discuss variable temperature optical properties of δ′-(BEDT-TTF)$_2$CF$_3$CF$_2$SO$_3$, we first investigate vibrational properties of the CF$_3$CF$_2$SO$_3^-$ anion itself. The theoretical calculations were performed with Gaussian 03,[5] using the standard split-valence 6-311+G(d) and the correlation-consistent aug-cc-pVQZ basis sets with the hybrid density functional (B3LYP). Room temperature infrared spectrum of the lithium LiCF$_3$CF$_2$SO$_3$ salt dispersed in KBr matrix has been measured using a Bruker Equinox 55 FT-IR spectrometer.

Figure S7 shows infrared absorption spectrum of LiCF$_3$CF$_2$SO$_3$ together with the absorption spectrum of the CF$_3$CF$_2$SO$_3^-$ anion calculated using DFT methods at the B3LYP/6–311+G(d) and B3LYP/aug-cc-pVQZ theory levels. Selected experimental and calculated frequencies together with the assignments are listed in Table S1.

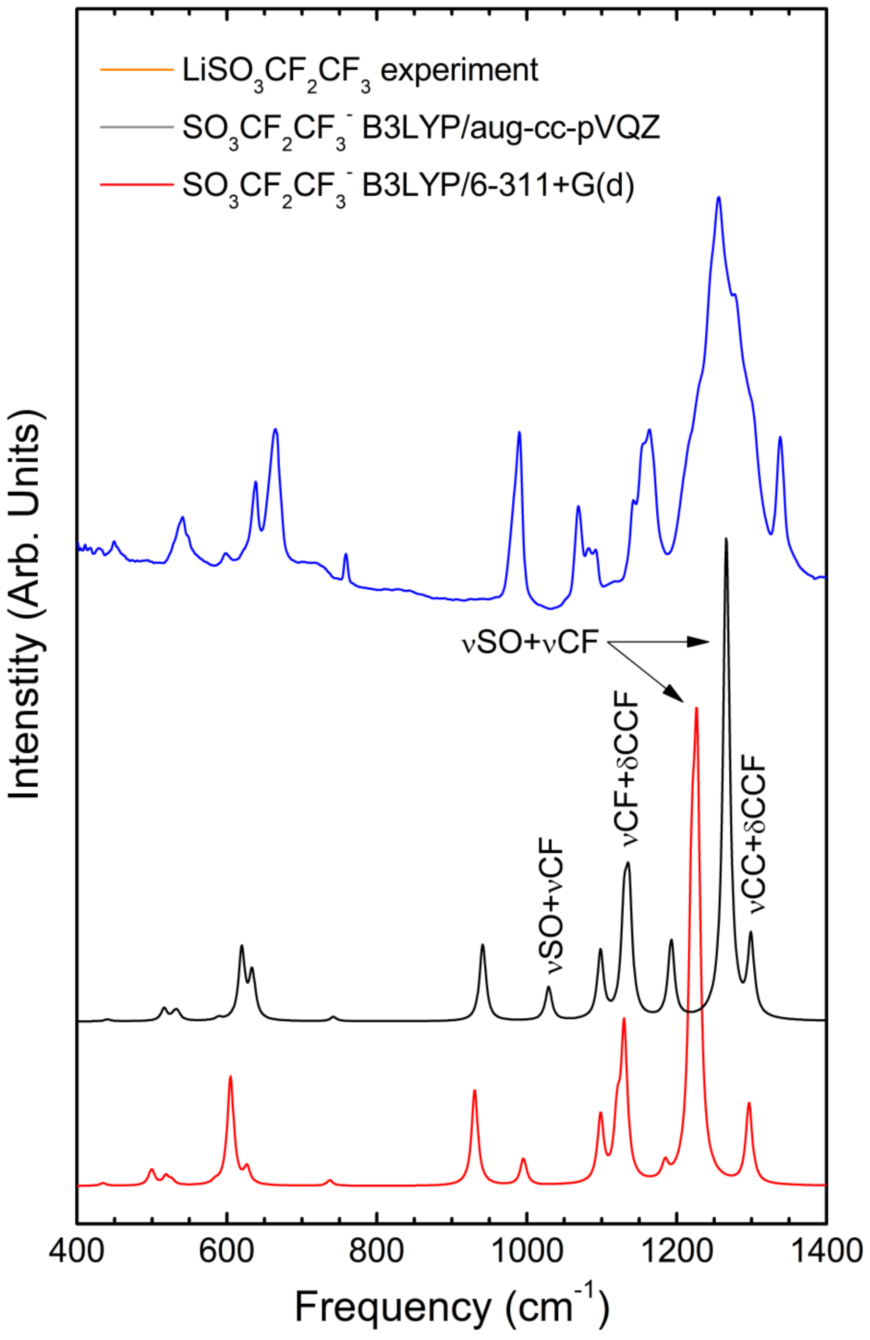

Figure S7. Experimental infrared absorption spectrum of $LiCF_3CF_2SO_3$ together with the absorption spectrum of the $CF_3CF_2SO_3^-$ anion calculated using DFT methods at the B3LYP/6–311+G(d) and B3LYP/aug-cc-pVQZ theory levels.

Table S1. The most intense infrared vibrational modes of the $CF_3CF_2SO_3^-$ anion.

$CF_3CF_2SO_3^-$ anion DFT calculations (B3LYP)				Experiment ($LiCF_3CF_2SO_3$)	Approximate description
6-311+G(d)		aug-cc-pVQZ			
Wavenumber (cm^{-1})	Intensity (km/mol)	Wavenumber (cm^{-1})	Intensity (km/mol)	Wavenumber (cm^{-1})	
1297	127	1299	123	1338	CC stretching + CCF bending
1227	609	1267	461	1256	SO stretching + CF stretching
1220	395	1265	328		SO stretching + CF stretching
1185	26	1193	123		CF stretching + SO stretching
1130	238	1136	183	1164	CF stretching + CCF bending
1121	94	1130	151		CF stretching + CCF bending
1098	105	1098	107	1142	CF stretching + CCF bending
996	42	1029	53	1069	SO_3 stretching sym. + CF stretching
931	152	941	121	990	CCF bending + CF stretching + SO_3 stretching sym.
737	9	742	7	759	CF_3 umbrella mode + SC stretching
627	26	634	73	638	CF_2 scissoring + CCF bending in CF_3 + CCS bending
605	172	620	112	664	SO_3 umbrella mode
526	7	530	8	541	FCF bending + OSO bending
519	14	534	13		OSO bending + FCF bending
499	25	516	20		OSO bending + FCF bending

SI3 Selected vibrational modes of *δ′*-(BEDT-TTF)$_2CF_3CF_2SO_3$ sensitive to 200 K phase transition

Structural phase transition at 200 K is related with charge ordering. Both the structural modifications and charge disproportionation of ≈ 0.2*e* influence vibrational modes of BEDT-TTF donor molecule observed in optical conductivity spectra of *δ′*-(BEDT-TTF)$_2CF_3CF_2SO_3$. In addition to the charge sensitive ν_{27} (B_{1u}) mode, splitting or activation below 200 K is observed for ν_{60} (B_{3g}) mode in *a*-direction as well as ν_{67} (B_{3u}), ν_{46} (B_{2u}), and ν_{29} (B_{1u}) modes in *c*-direction, as shown in Fig. S8. The mode assignment has been performed based on Ref. 6.

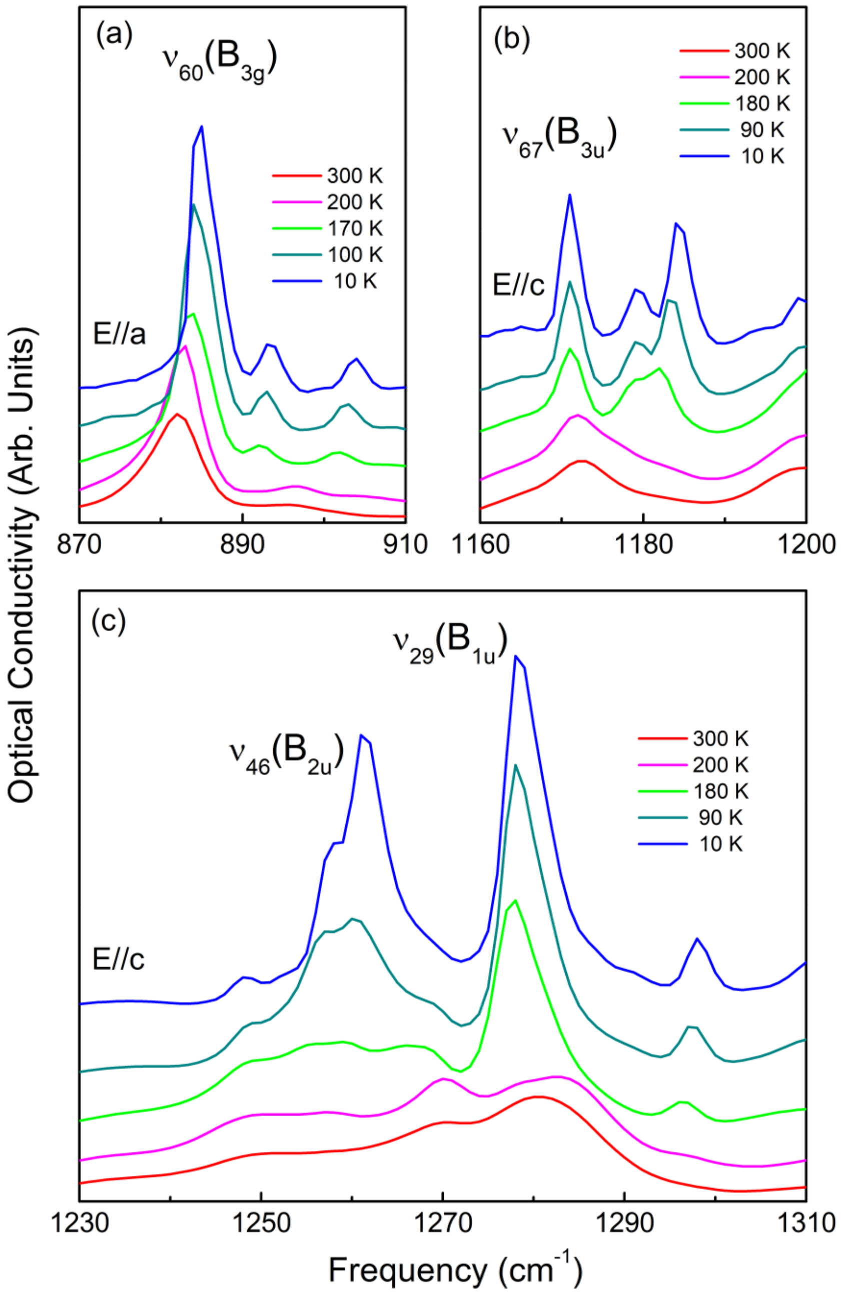

Figure S8. Temperature-dependent behavior of the selected vibrational modes observed in optical conductivity spectra of δ'-(BEDT-TTF)$_2$CF$_3$CF$_2$SO$_3$ polarized in the *a*- ((a) ν_{60} (B_{3g}) mode) and *c*-directions ((b) ν_{67} (B_{3u}) mode, and (c) ν_{46} (B_{2u}), ν_{29} (B_{1u}) modes).

References

1. Schlueter, J. A.; Ward, B. H.; Geiser, U.; Wang, H. H.; Kini, A. M.; Parakka, J.; Morales, E.; Koo, H.-J.; Whangbo, M.-H.; Winter, R. W. et al. Crystal Structure, Physical Properties and Electronic Structure of a New Organic Conductor β′′-(BEDTTTF)$_2$SF$_5$CHFCF$_2$SO$_3$, J. Mater. Chem. **2001**, *11*, 2008.

2. Ward, B. H.; Schlueter, J. A.; Geiser, U.; Wang, H. H.; Morales, E.; Parakka, J. P.; Thomas, S. Y.; Williams, J. M.; Nixon, P. G.; Winter, R. W. et al. Comparison of the Crystal and Electronic Structures of Three 2:1 Salts of the Organic Donor Molecule BEDT-TTF with Pentafluorothiomethylsulfonate Anions $SF_5CH_2SO_3^-$, $SF_5CHFSO_3^-$,and $SF_5CF_2SO_3^-$, Chem. Mater. **2000**, *12*, 343.

3. Schlueter, J. A.; Ward, B.; Geiser, U.; Mohtasham, J.; Winter, R.; Gard, G. Chemical Modification of the Superconducting β''-$(ET)_2SF_5CH_2CF_2SO_3$ Structure through Use of $CF_3CRR'SO_3^-$ Anions, Mol. Cryst. Liq. Cryst. **2002**, *380*, 129.

4. Guionneau, P.; Kepert, C. J.; Bravic, G.; Chasseau, D.; Truter, M. R.; Kurmoo, M.; Day, P. Determining the Charge Distribution in BEDT-TTF Salts. Synth. Met. **1997**, *86*, 1973–1974.

5. Gaussian 03, Revision D.01, Frisch, M. J.; Trucks, G. W.; Schlegel, H. B.; Scuseria, G. E.; Robb, M. A.; Cheeseman, J. R.; Montgomery, Jr., J. A.; Vreven, T.; Kudin, K. N.; Burant, J. C.; Millam, J. M.; Iyengar, S. S.; Tomasi, J.; Barone, V.; Mennucci, B.; Cossi, M.; Scalmani, G.; Rega, N.; Petersson, G. A.; Nakatsuji, H.; Hada, M.; Ehara, M.; Toyota, K.; Fukuda, R.; Hasegawa, R.J.; Ishida, M.; Nakajima, T.; Honda, Y.; Kitao, O.; Nakai, H.; Klene, M.; Li, X.; Knox, J. E.; Hratchian, H. P.; Cross, J. B.; Bakken, V.; Adamo, C.; Jaramillo, J.; Gomperts, R.; Stratmann, R. E.; Yazyev, O.; Austin, A. J.; Cammi, R.; Pomelli, C.; Ochterski, J. W.; Ayala, P. Y.; Morokuma, K.; Voth, G. A.; Salvador, P.; Dannenberg, J. J.; Zakrzewski, V. G.; Dapprich, S.; Daniels, A. D.; Strain, M. C.; Farkas, O.; Malick, D. K.; Rabuck, A. D.; Raghavachari, K.; Foresman, J. B.; Ortiz, J. V.; Cui, Q.; Baboul, A. G.; Clifford, S.; Cioslowski, J.; Stefanov, B. B.; Liu, G.; Liashenko, A.; Piskorz, P.; Komaromi, I.; Martin, R. L.; Fox, D. J.; Keith, T.; Al-Laham, M. A.; Peng, C. Y.; Nanayakkara, A.; Challacombe, M.; Gill, P. M. W.; Johnson, B.; Chen, W.; Wong, M. W.; Gonzalez, C.; and Pople, J. A.; Gaussian, Inc., Wallingford CT, 2004.

6. McGuire, J. J.; Rõõm, T.; Pronin, A.; Timusk, T.; Schlueter, J. A.; Kelly, M. E.; and Kini, A. M. Incoherent interplane conductivity of κ-$(BEDT\text{-}TTF)_2Cu[N(CN)_2]Br$, Phys. Rev. B **2001**, *64*, 094503.
